# Genetic analysis and QTL mapping for multiple biotic stress resistance in cassava

**DOI:** 10.1371/journal.pone.0236674

**Published:** 2020-08-05

**Authors:** Ana Luísa Garcia-Oliveira, Bernadetha Kimata, Salum Kasele, Fortunus Kapinga, Esther Masumba, Geoffrey Mkamilo, Caroline Sichalwe, Jessen V. Bredeson, Jessica B. Lyons, Trushar Shah, Satoru Muranaka, Manpreet S. Katari, Morag E. Ferguson

**Affiliations:** 1 International Institute of Tropical Agriculture (IITA), Nairobi, Kenya; 2 Excellence in Breeding (EiB), CIMMYT c/o ICRAF, Nairobi, Kenya; 3 Tanzania Agricultural Research Institute (TARI), Naliendele, Mtwara, Tanzania; 4 TARI, Ukiriguru, Tanzania; 5 TARI, Kibaha, Tanzania; 6 Molecular and Cell Biology Department, University of California, Berkeley, California, United States of America; 7 Japan International Research Center for Agricultural Sciences (JIRCAS), Tsukuba, Japan; 8 Department of Biology, New York University, New York, New York, United States of America; Shanghai Institutes for Biological Sciences, CHINA

## Abstract

In Sub-Saharan Africa cassava (*Manihot esculenta* Crantz) is one of the most important food crops where more than 40% of the population relies on it as their staple carbohydrate source. Biotic constraints such as viral diseases, mainly Cassava Mosaic Disease (CMD) and Cassava Brown Streak Disease (CBSD), and arthropod pests, particularly Cassava Green Mite (CGM), are major constraints to the realization of cassava’s full production potential in Africa. To address these problems, we aimed to map the quantitative trait loci (QTL) associated with resistance to CBSD foliar and root necrosis symptoms, foliar CMD and CGM symptoms in a full-sib mapping population derived from the genotypes AR40-6 and Albert. A high-density linkage map was constructed with 2,125 SNP markers using a genotyping-by-sequencing approach. For phenotyping, clonal evaluation trials were conducted with 120 F_1_ individuals for two consecutive field seasons using an alpha-lattice design at Chambezi and Naliendele, Tanzania. Previously identified QTL for resistance to CBSD foliar symptoms were corroborated, and a new putative QTL for CBSD root necrosis identified (*qCBSDRNc14AR*) from AR40-6. Two QTL were identified within the region of the previously recognized *CMD2* locus from this population in which both parents are thought to possess the *CMD2* locus. Interestingly, a minor but consistent QTL, *qCGM18AR*, for CGM resistance at 3 months after planting stage was also detected and co-localized with a previously identified SSR marker, NS346, linked with CGM resistance. Markers underlying these QTL may be used to increase efficiencies in cassava breeding programs.

## Introduction

Cassava (*Manihot esculenta* Crantz) is widely grown within the tropics of Africa, Asia, and Latin America where it is consumed as a food and utilized as an industrial crop primarily for starch [1]. The broad adaptation, resilience, and excellent source of calories makes cassava a popular choice for African farmers, but its productivity in Africa is far below that of its potential and that achieved in countries outside of the continent. Biotic stresses inflict a large yield penalty and impact the availability of clean planting material [2–4]. These stresses include viral diseases and insect pests, particularly Cassava Mosaic Disease (CMD), Cassava Brown Streak Disease (CBSD), and Cassava Green Mite (CGM) (*Mononychellus tanajoa*) [4,5]. Although yield losses due to individual biotic constraints vary from year to year, the combined effect of diseases and pests implies that there is potential for near total crop failure under field conditions [6,7]. Viruses that cause CMD and CBSD are spread by the whitefly vector, *Bemisia tabaci*, and via vegetative stem cuttings. From the 1930s, after the first detailed report on viruses that cause disease in cassava [8], about 15 specific virus species and several strains have been identified in Africa that cause substantial yield losses in different cassava producing regions [4].

Compared to CMD, CBSD received comparatively little attention until the end of 20^th^ century. This is partly because it was endemic to a relatively restricted area in the coastal lowlands of Kenya, Tanzania, and in areas along Lake Malawi in Mozambique and Malawi [9–11]. However, in the past decade it has also been reported at higher altitudes and inland of countries surrounding the Great Lakes regions, including Burundi [12], Rwanda [13], Uganda [7], and eastern DR Congo [14], as well as further afield such as in southern Sudan [15] and the islands off East Africa, including Mayotte Island [16]. In heavily affected regions, farmers have been forced to abandon cassava production [12,14,17], with significant reductions in storage root quality observed in susceptible cultivars and losses of 70% or more [18,19]. The spread of CBSD from East to West Africa is considered a major threat to food security [20].

Breeding for disease resistance continues to be one of the most reliable, sustainable, and cost-effective measures to overcome multiple disease and pest stresses [21]. The first attempts to select for cultivars resistant to CBSD were during the 1930’s at the former Amani Research Station (Tanzania). Studies on CBSD inheritance and gene action indicate that CBSD resistance is multigenic and recessive [22]. Using Mozambican parental lines, Zacarias [23] suggested that non-additive gene effects contribute to the control of CBSD resistance, results that diverged from ones obtained previously by Jennings [24], and later by Kulembeka *et al*. [25], that showed CBSD resistance in Tanzanian varieties seemed to be due to additive gene effects. Studies to identify QTL associated with resistance to CBSD foliar symptoms and root necrosis have been conducted using biparental populations derived from Tanzanian landraces [26,27] and a Ugandan breeding population [28]. Here we utilized a source of germplasm from South America where additional sources of resistance, and even immunity, to CBSD have recently been identified [29].

CMD is widely distributed and one of the most notorious viral diseases of cassava in Africa, resulting in an estimated annual loss of 34 million tonnes [30]. The disease is best kept under control by the deployment of resistant cultivars [31]. Following the establishment of the Root and Tuber Improvement Program in 1971 at International Institute of Tropical Agriculture (IITA), Ibadan, Nigeria, tremendous progress has been made in producing improved cassava cultivars with resistance to CMD [32]. Among the well-known sources of resistance to CMD (*CMD1*, *CMD2* and *CMD3*), *CMD2* has been extensively utilized in cassava breeding programmes in Africa and recently introduced in Asia particularly India and Thailand [31–33]. Despite numerous studies, causative gene(s) of the widely recognized *CMD2* locus [34] have not been identified. The exact nature of this resistance, whether monogenic [35, 36], polygenic or multi-allelic [26,37] or epigenetic [38] is still under debate, although significant new knowledge on the genomic organization of this locus has been provided by Kuon *et al*. [39]. Additional loci of smaller effect including *CMD1* [40] and *CMD3* explaining 11% of the phenotypic variance [41] have been recognized in different germplasm sources.

CGM is a notorious arthropod pest that seriously affects the cassava canopy (ability to stay green), particularly in dry seasons. CGM was originally described on cassava in Brazil [42], but occurs in almost all cassava growing regions, particularly in Brazil and Africa, where it causes losses of up to 51% [43,44]. Biological control and deployment of host-plant resistance are important control measures against CGM [45, 46]. *Typhlodromalus aripo*, a predatory mite introduced from Brazil, is the most effective agent for biological control of CGM in Africa [47], although its effectiveness is sometimes compromised by a failure to establish properly, either because of a lack of a suitable host cassava genotype and/or climatic conditions [48]. In the past, breeding efforts have mainly focused on indirect selection against CGM through selection for associated morphological traits such as pubescent leaves (PL), large compact shoot apices, enhanced leaf retention (LR) and stay green (SG) characteristics [49,50]. Little progress in understanding the inheritance of CGM resistance has been achieved through field studies, although additive gene action is thought to be important [48,51]. Recently, QTL for PL and LR were found within a 4- to 8-MB region on chromosome 8, and for PL and SG on chromosome 12. These co-localized with QTL for CGM severity at 6 MAP [50,52]. Using bulk segregant analysis (BSA), Macea-Choperena *et al*. [53] identified SSR markers NS1099 and NS346 as having the highest association with CGM resistance in individuals of different families.

In the present study, we aimed to construct a high-density linkage map through SNP genotyping and map QTL for field resistance to CBSD foliar and root necrosis, and CMD and CGM foliar symptoms using a full-sib population derived from a cross between an elite genotype, AR40-6 (source of CBSD, CMD and CGM resistance), and a Tanzanian local variety, Albert (CBSD and CGM susceptible and CMD resistant).

## Materials and methods

### Generation of mapping population

Among the cassava genotypes previously screened for viral diseases in Tanzania, AR40-6 was identified as having high levels of resistance to CMD, CBSD, and CGM and was thus selected for QTL mapping. AR40-6 was bred at the International Centre of Tropical Agriculture (CIAT), Colombia and has approximately 12.5% hybrid genome from wild species *M*. *esculenta* ssp. *flabellifolia*, 0.3% *M*. *glaziovii*–*M*. *esculenta* introgression segments [54], 50% CMD resistant variety C39 from IITA [55], 6.25% of Thai origin from Rayong 1 (via Rayong 60) [56] and the remainder approximately 30.25% of South American origin. AR40-6 was positively selected for the *CMD2* locus at CIAT using SSR markers. In contrast, Albert is a locally adapted variety popular in South-eastern Tanzania and is susceptible to CBSD and CGM but has good agronomic characters and resistance to CMD [57, 58]. It is a full-sib of TME117 [54] and was found to possess two putative QTL within the *CMD2* locus by Masumba *et al*. [26] in a bi-parental mapping population with the landrace Namikonga. To generate the full-sib F_1_ mapping population, crosses between AR40-6 (female) and Albert (male) were performed at Naliendele, Tanzania during 2009 according to a standard protocol [59]. A total of 1,015 F_1_ seeds were obtained and, after a flotation test to eliminate poor quality seed, they were germinated in seed trays on a bench under screen house conditions at the Tanzanian Agricultural Research Institute (TARI), Kibaha, Tanzania. The integrity of the F_1_ seedlings was tested using SSR markers. For clonal multiplication, only true F_1_ seedlings were planted in a low disease pressure area, isolated from other cassava plants at Makutupora, Tanzania in March 2010.

### Field evaluation for disease and pest resistance/tolerance

A total of 120 F_1_ individuals were phenotyped in clonal evaluation trials (CET) over two years (2013 and 2014) at two locations namely; Chambezi (5°54’S and 35°57’E at an altitude of 39m above sea level (asl)) and Naliendele (10°23’S and 40°09’E at an altitude of 137m asl) in Tanzania. Further details of agro-climatic and soil conditions are given in S1 Fig. An alpha lattice design was used with two replications. Each genotype was grown in single-row plots of five plants with 1 × 1 m spacing (row-to-row and plant-to-plant). To augment disease pressure, the trials were planted in December which coincides with the short rains and extremely high CBSD pressure in Chambezi [60]. In addition, stakes taken from plants close to the experimental sites that clearly exhibited CMD and CBSD symptoms were planted around the evaluation trials to increase disease pressure. Sufficient replication within trial, across sites and years was also used to reduce the effect of any escape from disease challenge. Foliar symptoms of CBSD, CMD, and CGM were evaluated on a subjective scale of 1 (no symptoms) to 5 (severe leaf symptoms with die back in the case of CBSD) at 3 and 6 months after planting (MAP) [26,59]. For CBSD root necrosis symptoms, roots were harvested 12 MAP and necrosis was scored both qualitatively on a 1 (no visible root necrosis) to 5 (very severe necrosis of affected roots) scale [26,61] and quantified through analysis of photographs using ImageJ software (http://rsbweb.nih.gov/ij/).

Phenotypic data was analysed by performing analysis of variance (ANOVA) using the General Linear Model (Proc GLM) procedure of SAS software (SAS Institute, Cary, NC, USA) including environment (e), genotype (g), genotype by environment interactions (gxe), and replication effects (r) in the model, and from which the components of variance were estimated. Broad sense heritability (h^2^bs) across environments was computed using the formula; h^2^bs = σ^2^_g_ + σ^2^_ge_/e + σ^2^_e_/er [62] and classified as suggested by Johnson and Robinson [63].

### Genotyping, data analysis and construction of a genetic linkage map

Total genomic DNA was extracted from young leaf tissue for all the F_1_ full sib family individuals and both parents, using a modified Dellaporta *et al*. [64] method. Genotyping-by-sequencing (GBS) and single nucleotide polymorphism (SNP) calling was carried out at the University of California, Berkeley and used to develop the consensus map reported by ICGMC [65]. In that study SNPs were called against v4.1 of the draft cassava reference genome sequence but hereere SNPs were re-called against version 5.1. A series of quality checks were performed on the data before calculating the linkages. Markers with InDels, tri- and tetra-allelic loci, loci with identical segregation patterns and any markers with more than 20% missing values, were removed before generating linkage groups (LGs). For JoinMap^®^4.1, SNP marker data were coded according to the CP option in JoinMap^®^ 4.1 manual for outcrossing species [66]. The threshold of fit was set to be >5.0 with groups defined according to the independence LOD and a recombination frequency <0.4. Segregation distortion was tested using the chi-square goodness-of-fit test in JoinMap. The marker order and distances were calculated using the Maximum Likelihood Mapping function with default settings and a genetic map was constructed using the Kosambi mapping function [67].

### QTL analysis

QTL analysis was performed on the mean scores of both replications for CBSD foliar (3 and 6 MAP), CBSD root necrosis, CMD foliar (3 and 6 MAP) and CGM at each environment using the QTL mapping software package Genetic Analysis of Clonal F_1_ and Double cross (GACD) version 1.1 [68]. Based on the actual number of identified alleles in the two parents and the actual number of identifiable genotypes in the clonal F_1_ progenies, each marker locus was classified into four categories as described for GACD [68]. Four marker categories *i*.*e*., ABCD, A = B, C = D and AB = CD were assigned with the assumption that A and B are two alleles from the female parent at the marker locus, and C and D are two alleles from the male parent at the same locus. Category ABCD represents polymorphism in both parents whereas categories A = B and C = D represent only female and male polymorphism, respectively. On the other hand, category AB = CD represents the same polymorphism pattern in both female and male parents, similar to an F_2_ population derived from two inbred parents in self-pollinated and cross-pollinated species. Any missing values of marker type were coded as XX. In order to declare QTL significance, LOD thresholds were determined using the empirical formula LOD = χ^2^_αp_(df)/2ln as described by Yu *et al*. [69]. Results are from the .QIC file which reports an adjusted phenotypic variance explained (PVE). A QTL was considered a major QTL if it accounts for more than 10% phenotypic variance [70].

### Gene annotation

To identify putative genes underlying the major consistent QTL, the position of markers of interest were first converted from v5.1 to v6.1 on the *M*. *esculenta* reference genome (http://phytozome.jgi.doe.gov/pz/portal.html). All the predicted genes in the specific QTL region were extracted from the physical map. GO enrichment was performed by Singular Enrichment Analysis (SEA) using the Comparative Arabidopsis Genome Resource (https://www.arabidopsis.org/). Over- and under-represented annotation-enrichment GO terms were extracted with default parameters and only pathways with *p*<0.5 values were considered.

## Results

One thousand and fifteen F_1_ seeds were sown, however a germination rate of 21.5% left 218 seedlings for establishment in the multiplication block. Of these, 18 and 41 seedlings were identified by SSR markers as self-fertilized and off-types, respectively. After evaluation, 159 true F_1_ genotypes including both parents were chosen for genotyping, but a high level of missing data in five samples reduced the number to 154.

### Disease and pest traits assessment, and their frequency distribution

**Fig 1:**
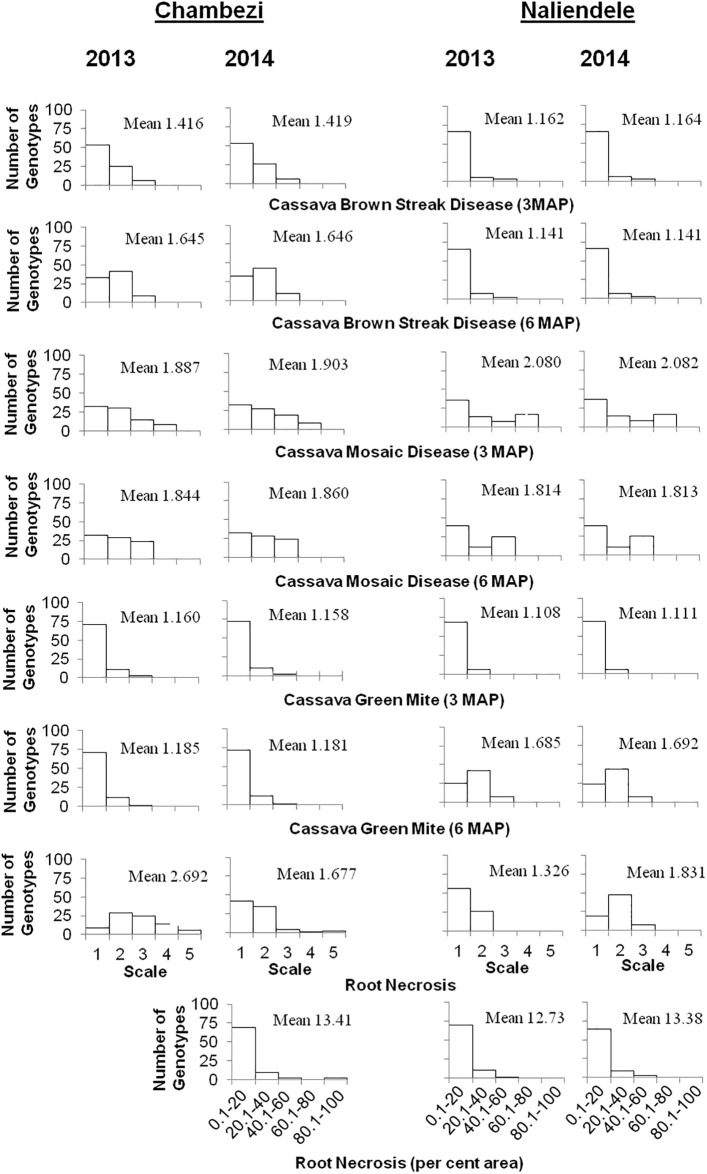
Frequency distribution of reaction of the full-sib families against CBSD, CMD, CGM and RN in clonal evaluation trials at Chambezi and Naliendele, Tanzania during cropping seasons 2013 and 2014. The foliar CBSD, CMD and CGM, and necrosis of root (RN), scores on a 1–5 scale from resistant to susceptible and root necrosis *per cent* (% area) are shown on the x-axis, and the number of genotypes (F_1_ families) with each score is plotted on the y-axis.

Typical symptoms of all the investigated traits were observed adequately among the 120 F_1_ clones planted in both years and locations in Tanzania. Analysis of variance (ANOVA) for phenotypic traits showed a significant difference for the environmental effect indicating the presence of substantial environmental variance (S1 Table). Higher mean scores of CBSD foliar symptoms at 3 and 6 MAP were observed in both years in Chambezi compared to Naliendele (Fig 1). Similarly, higher root necrosis was observed throughout 2013 at Chambezi, but no significant difference was observed for both mean score and *per cent* root necrosis between the locations in 2014. Similar levels of CMD and CGM were observed across locations throughout the growing period, except for CGM in Naliendele, where more severe damage was consistently observed at 6 MAP in both years. F_1_ clone reactions to each of CBSD, CMD, and CGM were strongly correlated between years at each location, whereas correlations between Chambezi and Naliendele locations were poor (S2 Table). Some clones that scored 5 for CBSD root necrosis had die back that led to the death of the plant. High skewness and kurtosis values revealed that traits were not normally distributed (S3 Table). In general, the frequency distribution of reaction of the family against CBSD foliar and root necrosis, and foliar CMD and CGM showed significant skewing toward the resistant parental mean (Fig 1). The higher magnitudes of phenotypic coefficients of variation (PCV) compared to their genotypic coefficients of variation (GCV) for all traits clearly underscore the role of environmental factors on the expression of the studied traits (S3 Table). Moderate to high PCV and GCV were observed for all the studied traits. The broad sense heritability estimates for foliar CBSD, CMD, CGM at 3 and 6 MAP varied from 24.70%– 42.91%, 75.16%– 76.79% and 21.29%– 36.84% across the environments, respectively. Similarly, heritability estimates of 25.12% and 33.26% were observed for CBSD root necrosis if the traits were quantified on the basis of scale (1–5) or *per cent* root necrosis (S3 Table).

### Construction of linkage map

**Fig 2:**
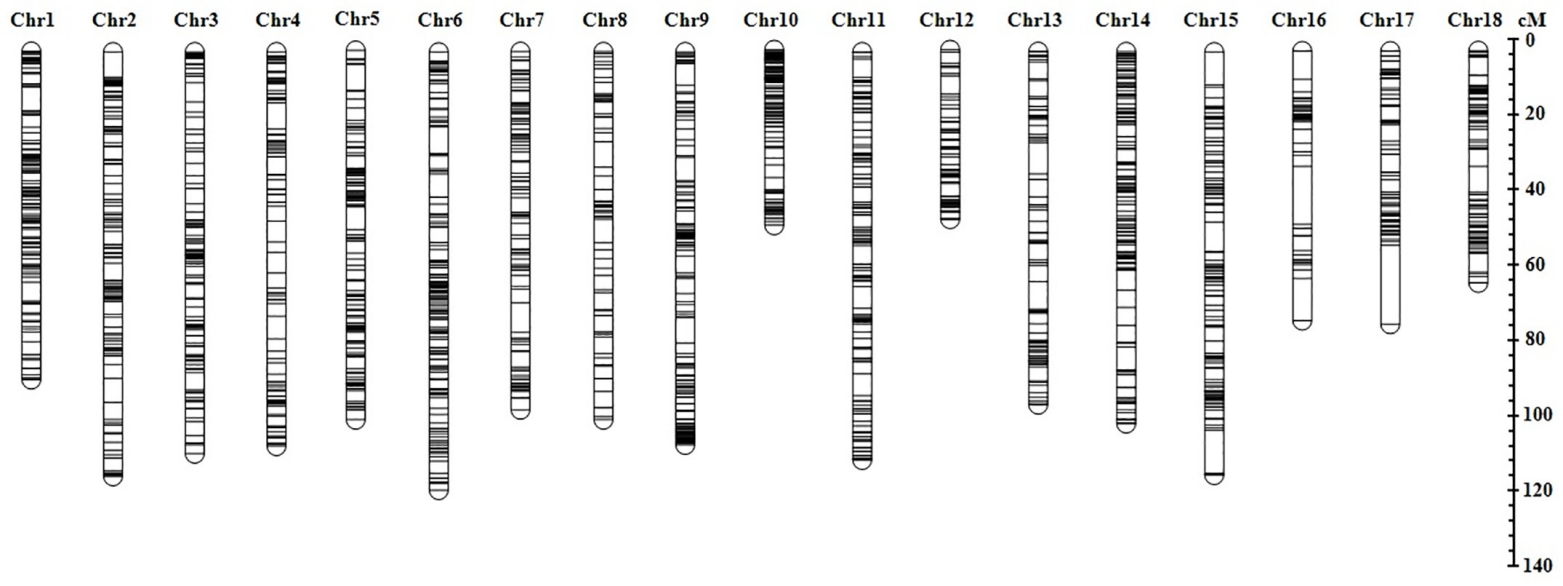
Schematic representation of genetic linkage map developed from full-sib cassava mapping population using 2,125 SNP markers. Horizontal bars on each chromosome represent mapped SNP markers and the scale bar to the right shows the lengths of linkage groups (Chromosomes) as measured in Kosambi cM. A more detailed map with marker names (*Manihot esculenta* v5.1 reference genome) and their position is presented in S2 Fig.

A total of 3,491 high-quality SNPs were generated by GBS from 147 genotypes. Following the stringent filtering of polymorphic SNPs (≥20% missing values; ≤5% MAF), 41 loci were deleted because they segregated in exactly the same way as another marker; furthermore, one marker was discarded for being tetra-allelic and 18 were discarded for being tri-allelic which left a total of 2,125 SNP markers (S4 Table). Prior to building the final genetic linkage map, 17 genotypes that > 20% missing data were also deleted. The resulting genetic map consisted of 2,125 markers on 18 linkage groups, representing the 18 chromosomes of cassava (2n = 36) with a length of 1730 cM (Fig 2; S4 Table; S2 Fig). Linkage group (LG) length varied from 49.8 cM (Chr. XII) to 121.9 cM (Chr. VI), with an average marker interval length between 0.45 cM to 1.71 cM per chromosome. The highest number of markers was mapped on chromosome VI (161), while the lowest number of markers was mapped to chromosome XVI (45). Overall, the map contained an average of 118 markers per linkage group with an average marker interval length of 0.81 cM.

### QTL mapping

To define a genome-wide LOD significance threshold, LOD scores were computed by performing permutation analysis with 1000 iterations. This corresponded to a threshold of LOD 3.25 to 8.49 for the studied traits. The high LOD threshold values are the result of the non-normally distributed phenotypic data, which are highly skewed towards resistance. Therefore, a QTL with a LOD score above 3.0 was considered significant [71]. QTL detected in at least two of the four environments were considered to be consistent [72], because QTL × environment interaction effects were highly significant and no QTL was consistently identified in all the four environments except a QTL for CGM at 3 MAP stage. Common names were assigned to consistent QTL associated with a specific disease or pest if it was detected at different stages (3 MAP or 6 MAP) and mapped to the same, or at least overlapping, genomic regions/marker intervals. A total of 17 consistent QTL were detected on chromosomes V, VIII, IX, X, XI, XII, XIII, XIV, XVI, and XVIII that each explained from 1.49% to 29.03% of phenotypic variation for the studied traits. A summary of these QTL, including their positions, LOD scores, genetic effects [additive effects of female (aF) and male (aM), and dominance effect d], and the adjusted PVE are shown in Table 1. Favourable alleles at each of the flanking markers are given in S5 Table.

**Table 1:** Stable QTL detected for field disease and pest resistance in the AR40-6 x Albert mapping population.

Trait	Scoring/ analysis stage	QTL	Chr.	P (cM)	Flanking Markers	Environment	LOD	Genetic effects			PVE (%)
								aF	aM	d	
CBSD-Foliar	*3 MAP*	*qCBSDFc11AR*	XI	10	18472436–18700194	NAL13	3.27	0.107	0.023	0.118	1.65
				10		NAL14	3.27	0.108	0.024	0.119	1.43
	*6 MAP*	*qCBSDFc18AR*	XVIII	57	10068641–10924641	CHA13	3.21	0.036	0.158	0.091	11.98
				57		CHA14	3.35	0.060	0.194	0.061	13.63
CBSD-	*Harvesting*	*qCBSDRNSc14AR*	XIV	63	12107960–13964594	NAL13	6.38	0.221	0.162	0.220	8.72
Necrosis*				67		NAL14	3.86	0.022	-0.165	0.045	8.18
				65		CHA14	7.12	-0.428	0.478	-0.525	8.61
CBSD-	*Harvesting*	*qCBSDRNAc8AR*	VIII	79	4660806–5883289	NAL13	3.60	-3.568	-3.838	3.892	9.70
Necrosis**				75		CHA14	7.87	-9.287	-8.126	10.556	2.77
		*qCBSDRNAc14AR*	XIV	62	12866832–13596996	NAL13	3.88	4.327	4.677	4.169	2.25
				60		NAL14	6.44	-4.878	-4.157	4.878	9.37
				65		CHA14	7.03	-10.038	10.821	-9.469	2.45
		*qCBSDRNAc18AR*	XVIII	25	6433344–6501916	NAL13	11.49	5.718	4.944	4.907	3.05
				25		CHA14	6.68	12.517	12.418	13.418	2.13
CMD	*3 MAP*	*qCMDc9AA*	IX	98	16028318–16697056	CHA13	3.04	-0.247	-0.066	0.132	10.60
				98		CHA14	5.45	-0.208	-0.026	0.009	7.13
		*qCMDc10*.*1AA*	X	20	3562661–3711657	NAL13	3.70	-0.279	-0.080	0.012	6.65
				20		NAL14	3.68	-0.280	-0.079	0.011	6.70
		*qCMDc12*.*1AA*	XII	22	5237169–5330694	NAL13	7.73	0.271	-0.064	-0.395	17.85
				22		NAL14	7.67	0.272	-0.064	-0.394	17.77
				22		CHA13	6.47	0.127	-0.110	-0.356	17.83
	*6 MAP*	*qCMDc10*.*2AA*	X	15	16727950–17833540	CHA13	3.67	0.111	0.042	-0.348	10.72
				15		CHA14	4.16	0.109	0.028	-0.290	8.77
		*qCMDc12*.*2AA*	XII	18	5224029–6745505	NAL13	24.00	0.025	-0.446	0.013	41.12
				18		NAL14	32.79	0.013	-0.486	0.020	45.97
		*qCMDc12*.*1AA*	XII	22	5237169–5330694	NAL13	4.15	0.044	-0.019	-0.183	7.33
				22		NAL14	7.36	0.017	-0.038	-0.178	7.64
				28		CHA14	4.16	0.084	-0.103	-0.243	3.47
CGM	*3 MAP*	*qCGMc5AR*	V	95	1801930–2287186	NAL13	7.24	-0.207	0.150	-0.220	1.74
				95		NAL14	4.21	-0.167	0.106	-0.172	2.66
				95		CHA13	3.94	-0.131	0.224	-0.223	6.38
		*qCGMc9AR*	IX	4	984855–1938190	NAL13	6.97	-0.156	0.147	-0.138	1.94
				4		NAL14	3.34	-0.151	0.140	-0.141	2.81
				4		CHA13	3.39	-0.318	0.324	-0.337	7.44
				4		CHA14	5.69	-0.387	0.363	-0.367	1.76
		*qCGMc13AR*	XIII	85	16122724–16635048	CHA13	9.49	-0.301	-0.366	0.305	11.06
				85		CHA14	11.96	-0.371	-0.415	0.349	1.82
		*qCGMc18AR*	XVIII	11	3265545–3701705	CHA13	4.28	0.253	-0.268	-0.287	8.60
				11		CHA14	4.37	0.221	-0.194	-0.221	1.35
	*6 MAP*	*qCGMc16AR*	XVI	19	15707994–1619079	CHA13	6.93	-0.261	0.280	-0.258	7.19
				19		CHA14	6.81	-0.264	0.273	-0.262	7.90

Chr.: Chromosome number; P: Position; LOD: Logarithm of odds, aF and aM: additive effects of Female and Male, respectively; d: dominance effect; PVE: Phenotypic variance explained; QTL name designated as described by Masumba *et al*. [29]; Phenotype data nomenclature was named in combination of the location and year such as Naliendele (NAL) and Chambezi (CHA), and the year of 2013 (13) and 2014 (14). SNP marker names are as per *Manihot esculenta* v5.1 reference genome. *and ** denote CBSD root necrosis assessed based on visual scale (1–5) and actual (%) necrotic area quantified by ImageJ program, respectively.

### QTL for CBSD foliar symptoms

QTL analysis for CBSD foliar resistance at 3 MAP identified one minor but consistent QTL (*qCBSDFc11AR*) on chromosome XI accounting for 2.5% phenotypic variation (Table 1). The peak position of *qCBSDFc11AR* was mapped at 10 cM in Naliendele 2013 and 2014 environments. Similarly, one consistent but major QTL (*qCBSDFc18AR*), explaining 12.87% of the phenotypic variation for CBSD foliar resistance at 6 MAP stage, was identified on chromosome XVIII in both years in Chambezi (Table 1). None of the QTL consistent across years for CBSD foliar resistance were found to be consistent across both 3 and 6 MAP stages.

### QTL for CBSD root necrosis

QTL analysis of CBSD root necrosis data obtained by two different methods revealed four consistent QTL on three different chromosomes (VIII, XIV, and XVIII) (Table 1). A single consistent QTL (*qCBSDRNSc14AR*), with LOD values ranging from 3.86 to 7.12 and contributions to phenotypic variance of 8.18%– 8.72% was detected for root necrosis when the trait was quantified on the basis of visual score (1–5 scale). Contrarily, when root necrosis was quantified as *per cent* root necrosis area using ImageJ software, three consistent QTL (*qCBSDRNAc8AR*, *qCBSDRNAc14AR*, and *qCBSDRNAc18AR*), accounting for 2.13% to 9.70% phenotypic variation, were detected at chromosome(s) VIII, XIV, and XVIII with mean LOD values ranging from 3.6 to 11.49. Among these QTL, *qCBSDRNSc14AR/qCBSDRNAc14AR* were detected consistently with different quantification methods for root necrosis. However, all the QTL for root necrosis exhibited PVE less than 10% and were considered as minor QTL.

### QTL for CMD

**Fig 3:**
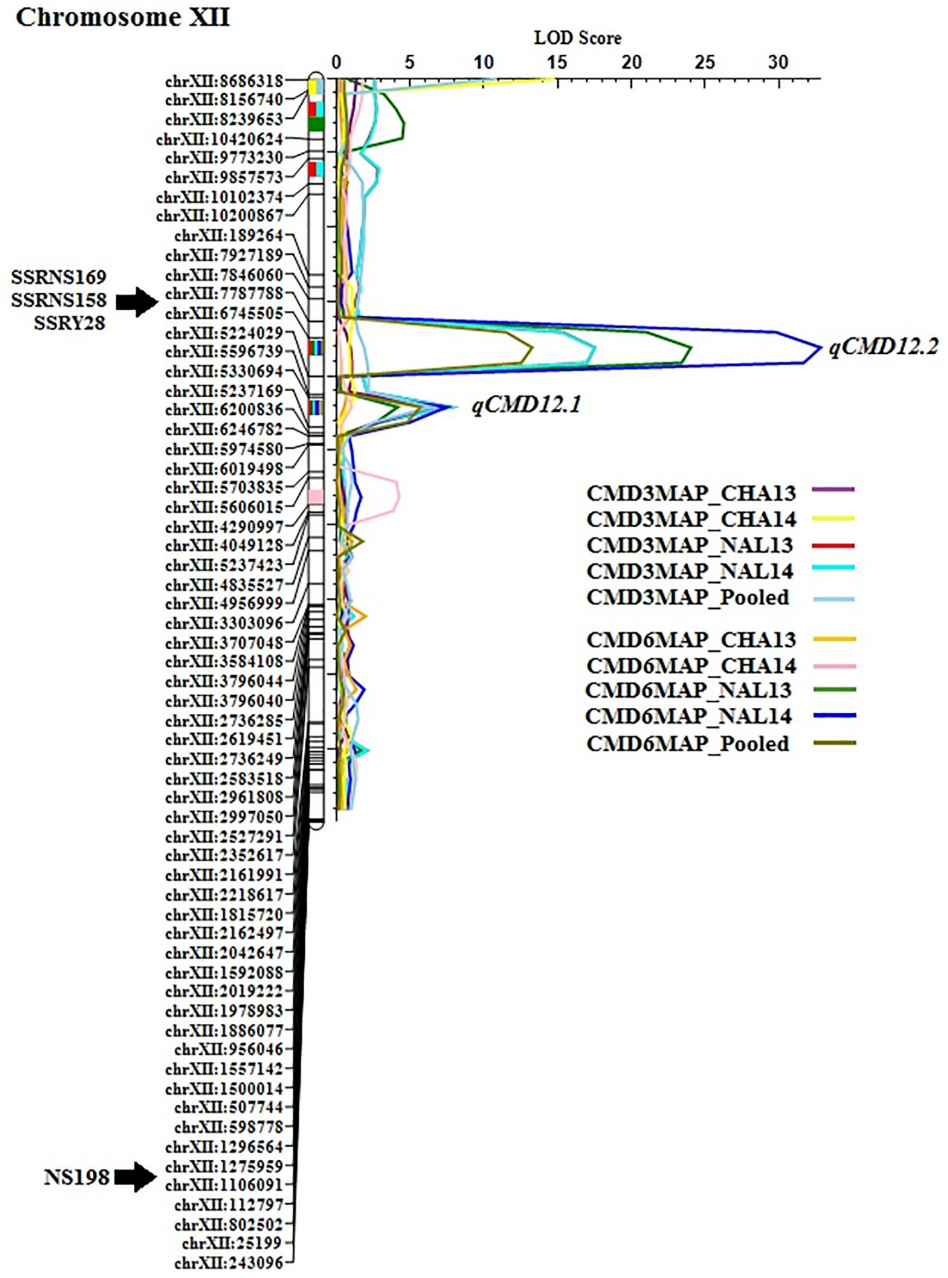
Chromosome XII linkage map showing the position of QTL identified in the present investigation in relation to the previously reported SSR markers; SSRY28, SSRNS158 and SSRNS169 (*CMD2*) and NS198 (*CMD3*) associated with CMD resistance loci in cassava. Trait nomenclature was named in combination of the disease, stage, location and year such as Cassava Mosaic Disease (CMD), 3 or 6 months after planting (3 MAP or 6 MAP) stage, crop season 2013 or 2014 at location Naliendele (NAL13 or NAL14) and Chambezi (CHA13 or CHA14), whereas pooled represents average of the trait from four environments either at 3 or 6 MAP stage.

QTL analysis for CMD data at both 3 and 6 MAP stages revealed a total of six putative QTL on chromosomes IX, X, and XII accounting for 3.47%– 45.97% PVE (Table 1). At 3 MAP, QTL analysis identified three consistent QTL (*qCMDc9AA*, *qCMDc10*.*1AA*, and *qCMDc12*.*1AA*), among them a major QTL, namely *qCMDc12*.*1AA* located at position 22 cM between markers cXII:5237169 and cXII:5330694 on chromosome XII, showed the highest contribution (19.09% PVE) to the variance (Fig 3). Similarly, three consistent QTL (*qCMDc10*.*2AA*, *qCMDc12*.*1AA*, and *qCMDc12*.*2AA*) were also detected for CMD resistance at 6 MAP stage. Of these QTL, *qCMDc12*.*1AA* and *qCMDc12*.*2AA* were located in adjacent regions on chromosome XII with peak positions at 22 cM and 18 cM, respectively. QTL *qCMDc12*.*2AA* for CMD resistance detected at position 18 cM between markers cXII:5224029 and cXII:6745505 on chromosome XII exhibited the highest LOD score up to 32.79 with PVE ranging from 41.12%– 45.97% (numbers after the chromosome designation cXII refer to the SNP base pair position on v5.1 of the cassava reference genome sequence. The sequence of markers on the genetic linkage map is not consistent with that on v5.1 of the physical map). Only major QTL *qCMDc12*.*1AA* was co-localized at both 3 and 6 MAP stage. It is worth noting that although *qCMDc12*.*1AA* and *qCMDc12*.*2AA* are sequentially located to one another at 22 cM and 18 cM on the genetic linkage map, *qCMDc12*.*2AA* encompasses *qCMDc12*.*1AA* on the physical map as flanking markers are cXII:5224029 and cXII:6745505, and cXII:5237169 and cXII:5330694, respectively. On the latest version of the physical map (v6.1), *qCMDc12*.*1AA* is positioned between c12:5530061 bp and c12:5770027 bp and *qCMDc12*.*2AA* between c12:5543126 bp and c12:7460818, so there is minor overlap in these QTL.

### Comparison of CMD chromosome 12 QTL with previously mapped markers

To more accurately compare the relative positions of SNP markers associated with QTL found in this study with those previously identified, all relevant markers were located on the *M*. *esculenta* v6.1 reference genome (http://phytozome.jgi.doe.gov/pz/portal.html) (S6 Table). In the present investigation, *qCMDc12*.*1AA* is located just outside the range of previously mapped markers with QTL *qCMDc12*.*2AA* [5,543,126–7,460,818 bp (v6.1)] encompassing the secondary peak from Wolfe *et al*. [37] at 7,032,174 bp (v6.1), Rabbi *et al*. [35] (S5214.30911) at 6,305,022 bp, Rabbi *et al*. [36] (S5214.780931) at 6,911,164 bp and overlapping with a peak of Masumba *et al*. [26] *qCMDc12*.2A [6,648,605–8,645,322 bp (v6.1)]. Previously described SSR markers associated with CMD resistance (SSRNS169, SSRNS158 and SSRY28) were identified in the interval 7,541,052–7,731,970 bp (v6.1), just outside the range of *qCMDc12*.*2AA* (Fig 3). This confirmed the co-localization of *CMD2* locus with *qCMDc12*.*2AA*. SSR marker NS198, associated with the *CMD3* locus, mapped to interval cXII:1106091 –cXII:1275959 on chromosome XII which was found approximately 30.3 cM from the *CMD2* locus (SSRY28). In the *M*. *esculenta* v6.1 reference genome, the positions of the markers defining *CMD2* and *CMD3* loci confirmed that *CMD3* locus is located further towards the beginning of the chromosome than the *CMD2* locus on chromosome XII.

### QTL for CGM

A total of five consistent QTL were detected for CGM resistance at 3 and 6 MAP (Table 1). Of these, four QTL (*qCGMc5AR*, *qCGMc9AR*, *qCGMc13AR*, and *qCGMc18AR*) for CGM resistance were identified at 3 MAP on chromosomes V, IX, XIII, and XVIII accounting for 1.35% to 11.06% phenotypic variance. Noticeably, QTL *qCGMc9AR*, explaining phenotypic variance between 1.76% to 7.44% was the only one consistently detected in all four environments. At 6 MAP only one QTL, *qCGMc16AR*, was detected for CGM resistance and accounted for phenotypic variance of 7.27% and had a LOD value of 2.59 across the years at Chambezi. All the QTL for CGM resistance were either specific to 3 or 6 MAP.

### Comparison with previously linked molecular markers for CGM and putative candidate genes in the QTL region

Of the three SSR markers previously reported for CGM resistance (NS346, NS1009, and NS1099) [53,73], markers NS346 and NS1099 mapped to chromosome XVIII, whereas the position of marker NS1009 was not confirmed on the *M*. *esculenta* v6.1 reference genome because only the forward primer could be located on chromosome XIV (S6 Table). Marker NS346 was found to co-localize with the minor QTL *qCGMc18AR* detected here. A total of 68 putative genes were present in the QTL *qCGMc18AR* region located between markers cXVIII:3701705 and cXVIII:3265545 (0.524 Mbp region) (Table 1; S7 Table) and included gene families of specific interest, such as receptor-like kinases (RLK) and transcription factors, particularly three defense-related leucine-rich repeat (LRR) RLK genes.

## Discussion

The viral diseases CBSD and CMD together cause over US$1 billion losses in annual production of cassava, and most of these losses affect smallholder farmers growing cassava as a food staple and selling surplus for income generation [74]. Although CMD and CBSD often occur in the same field, and co-infections do occur, there is no evidence for synergistic interaction between the viruses that cause CMD and CBSD [7,31,75]. The impact of CGM is more variable, being much more severe in dry seasons, but has not been widely quantified. One of the most effective means of combating these biotic constraints is the development of multiple disease and pest resistant varieties, in addition to biological control for CGM [21,76]. The identification of QTL associated with resistance would facilitate marker-assisted selection (MAS), which would enable selection for these traits at the seedling stage, dramatically reducing population sizes and enhancing efficiencies in breeding. In addition, this would enable pre-emptive breeding for CBSD resistance in West Africa, by applying artificial selection in the absence of disease pressure.

During the development of the mapping population, seed set was good, but germination was poor. Although seeds were stored for several months to break dormancy and flotation was used to eliminate non-viable seed, germination was only 21.5%. Poor germination was attributed to diurnal fluctuations in soil temperature as seeds were sown in pots on a bench. When pots were placed at ground level, germination increased. It is recommended that seed is germinated in seed beds, rather than in pots [27].

In the present investigation, substantial segregation was observed for reaction to CBSD foliar and root necrosis, and foliar CMD and CGM, indicating wide genetic variability among the full-sib family population (Fig 1, S3 Table). None of the progeny, however, exhibited immune responses to the diseases and pest under field conditions when considering performance across all the test environments. The skewed frequency distribution of all the studied traits observed toward the resistant phenotype could be due to the fact that extreme susceptibles succumbed to the disease(s) in the first few months after planting, and/or indicate that inheritance is oligogenic, as has been found in other host-pathogen systems including pest and viral disease resistance in cassava [27], and/or be due to environmental variation (moderately low or extremely higher disease/pest pressure) under field conditions [77]. This discrepancy may influence the ability to identify consistent QTL across multiple environments and becomes much more complex if the casual organism has multiple strains such as CBSD and CMD [26].

GBS has enabled a large number of SNP markers to be identified in a cost-effective manner and has facilitated the construction of high-density linkage maps in numerous crops, including cassava [35,36]. Here, stringent filters were used to generate high-quality SNPs, meaning that no imputation was necessary, although the number of markers was less than in other studies [35,36].

### QTL associated with tolerance to CBSD foliar symptoms

A minor but consistent QTL (*qCBSDFc11AR*) accounting for between 1.43%–1.65% phenotypic variation for foliar CBSD resistance at 3 MAP stage was detected on chromosome XI (18.4Mbp– 18.7Mbp). Nzuki *et al*. [27] detected *qCBSDRNFc11KR* associated with both root and foliar symptoms on the same arm of this chromosome but at a slightly different location (15.6–15.7Mbp). Kawuki *et al*. [28] reported five significant SNPs associated with CBSD root necrosis resistance on chromosome XI covering 3.88 Mbp between 19,872,319 to 23,751,929 bp that explained 14.6% of the phenotypic variation, but these SNPs failed to reach the genome-wide Bonferroni significance threshold. The right arm of chromosome XI thus appears to be implicated in tolerance to both CBSD root necrosis and foliar symptoms, although with relatively small effect. In accordance with this study, Nzuki *et al*. [27] also observed QTL for CBSD foliar symptoms on chromosome XVIII (*qCBSDFc18Ka* and *-b*), albeit at slightly different positions, at 5.3–5.4 Mbp and 5.7 to 6.1 Mbp, as opposed to 10.1–10.9 Mbp in this study. It thus appears that chromosome XVIII has QTL influencing both foliar and root necrosis.

### QTL associated with tolerance to CBSD root necrosis

Root necrosis was scored using two different methods; a subjective scale of 1–5 (RNS), and a quantitative method through the measurement of the percentage area of browning (RNA). Both methods identified a QTL on the right arm of chromosome XIV in three of the four environments (*qCBSDRNSc14*/*qCBSDRNAc14*). In addition, RNA identified two additional QTL on chromosomes VIII and XVIII, indicating greater resolution of this method of phenotyping. The QTL on chromosome XIV has so far not been detected by any other study [26–28], although Masumba *et al*. [26] reported a QTL associated with foliar resistance on the left arm of the same chromosome. Locus *qCBSDRNSc14*/*qCBSDRNAc14* could represent a new source of tolerance to CBSD root necrosis in AR40-6.

### CBSD root and foliar symptoms mainly influenced by different loci

In the present investigation, we assessed foliar CBSD symptoms at 3 and 6 MAP stages, and CBSD root necrosis on the basis of visual scale (1–5) as well as *per cent* root necrosis area by ImageJ program [78]. Two loci (*qCBSDFc11AR* and *qCBSDFc18AR*) for foliar CBSD and three genomic regions (*qCBSDRNAc8AR*, *qCBSDRNSc14AR/qCBSDRNAc14AR* and *qCBSDRNAc18AR*) for root necrosis were detected. This suggests that resistance to CBSD root necrosis is largely under different genetic control to CBSD foliar symptoms. It has also been observed in field conditions that foliar symptoms and root necrosis are often not highly correlated, as some varieties with clear leaf symptoms may fail to show root symptoms, while others that do not express leaf symptoms may produce root symptoms [6]. This is also consistent with the findings of Masumba *et al*. [26]. Similarly, Nzuki *et al*. [27] also reported that most of the QTL were associated with either root necrosis or foliar symptoms, except QTL *qCBSDRNFc11KR* and *qCBSDRNFc15K*, which were consistent across root and foliar symptoms, supporting the notion that resistance to foliar and root symptoms of CBSD are largely under different genetic control.

### QTL associated with tolerance to CMD

The first analysis of CMD resistance in an F_1_ population derived from a cross between the Nigerian landrace TME 3 (CMD resistant) and an improved line TMS 30555 (CMD susceptible) suggested Mendelian inheritance was governed by a single dominant locus designated as *CMD2* and tagged with SSR marker SSRY28 [34]. Subsequently, *CMD2* was confirmed in another Nigerian landrace TME 7 [79]. Now several molecular markers tightly linked with *CMD2* have been reported [26,34–37,41,79,80], although the designation of linkage group was different; group R [34], 8 [79], 16 [35,36] and chromosome XII [26].

In recent years, as larger population sizes have been used, coupled with higher density markers, it appears that two closely linked loci may be influencing CMD resistance, or this phenomenon may result from a multi-allelic effect. Two closely linked loci have been reported by Wolfe *et al*. [37] and Masumba *et al*. [26], and are also detected here (*qCMD12*.*1AA* and *qCMD12*.*2AA*). Large sections of repetitive DNA found within the CMD2 locus by Kuon *et al*. [39] could explain this.

In the present investigation, *qCMDc12*.*1AA* is located just outside the range of previously mapped markers, while the range of QTL *qCMDc12*.*2AA* encompasses the secondary peak found by Wolfe *et al*. [37] and both markers of Akano *et al*. and Rabbi *et al*. [34,35] and overlapping with a peak of Masumba *et al*. [26]. Previously described SSR markers associated with CMD resistance (SSRNS169, SSRNS158, and SSRY28) were identified just outside the range of *qCMDc12*.*2AA* but confirms the co-localization of *qCMDc12*.*2AA* with the *CMD2* locus (Fig 3). *qCMDc12*.*1AA* was the most stable and effective QTL, accounting for 17.77%–17.85% and 3.47%–7.64% of the phenotypic variation at 3 and 6 MAP stages, respectively. It was detected between markers cXII:5237169 and cXII:5330694 (Fig 3). Besides the *CMD2* locus, the position of SSR marker NS198 linked with the *CMD3* locus [41] was identified in interval cXII:1106091 –cXII:1275959 on v5.1 on the draft cassava genome sequence and was not found to be associated with any QTL on chromosome XII in this study (Fig 3).

Interestingly, both parents used in this study are thought to possess the dominant CMD2 locus, yet segregation in the F1 progeny was still observed. The *CMD2* locus was mapped in Albert by Masumba *et al*. [26], and AR40-6 was selected for the *CMD2* locus using SSR markers at CIAT. Wolfe *et al*. [37] found that the CMD2 locus accounted for between 30 to 66% of genetic resistance and Rabbi *et al*. [36] found that a single marker within the CMD2 locus accounted for 74% of the variation. Wolfe *et al*. [37] and Masumba *et al*. [26], also found, in addition to the major QTL in the vicinity of the CMD2 locus, a number of other QTL of small effect, distributed across many chromosomes. Likewise, other influencing QTL such as qCMDc10.2AA and qCMDc9AA in Chambezi and qCMDc10.1AA in Naliendele were found in this study. Although the CMD2 locus clearly has a large effect, and thus explains the skewness observed in CMD at 3 and 6 MAP, it does not explain all the variation, hence possibly explaining some of the segregation observed.

Two peroxidases (Manes.12G076200 and Manes.12G076300), a class of protein implicated in whitefly-mediated geminivirus infection in tomato [81], were identified in both CMD resistant and susceptible varieties at the *CMD2* locus defined in part by the major QTL of Wolfe *et al*. [37] and Kuon *et al*. [39]. Manes.12G076300 encodes a protein disulfide-isomerase-like 2–3 (PDI) which catalyzes the correct folding of proteins and prevents the clustering of precursors. In addition, a similar protein disulfide-isomerase-like 2–2 ortholog, a thioredoxin (PDIL2-2) was found within the secondary GWAS peak found by Wolfe *et al*. [37] and by Kuon *et al*. [39]. In barley, an ortholog of PDIL2-2 was identified as a major virus susceptibility factor with loss of function contributing resistance to bymoviruses [82]. Under this peak a Ubiquitin-conjugating enzyme E2 ortholog (UBC5) gene was also identified. Similar genes in the ubiquitinylation pathway have been found to influence plant virus infection response [37,83]. Kuon *et al*. [39] also highlight the possible involvement of a Suppressor of Gene Silencing 3 (SGS3) gene, 1.71 Mb downstream of the *CMD2* locus. They also noted that two genes present in the variety TME3 (containing CMD2 resistance elements) were missing in two CMD susceptible genotypes AM560-2 and 60444. These were both short gene models of unknown functions.

### QTL associated with tolerance to CGM

SSR markers NS1009 and NS346 have been reported for utilization in MAS for CGM resistance at CIAT as part of validation studies [73]. Macea-Choperena *et al*. [53] also displayed the association of previously reported marker NS346 and new marker NS1099 for CGM resistance in the individuals of different families of cassava. In the present investigation, a total of five consistent QTL for CGM resistance were identified, but all these QTL showed minor effects and were specific to either 3 or 6 MAP stages. Interestingly, marker NS346 linked with CGM resistance was found in the QTL *qCGMc18AR* region (cXVIII:3265545 –cXVIII:3701705) that accounted for a maximum of 8.6% of phenotypic variance for CGM resistance at 3 MAP stage. In addition, QTL *qCGMc9AR* located on chromosome IX accounted for between 1.76%– 7.44% of PVE and was the only QTL that was consistently detected in all four environments (Table 1).

In order to protect from disease and pest attack, plants evolved a wide range of defense mechanisms such as alteration in metabolic pathways and signaling molecules which stimulate defense-related processes [84]. The minor QTL *qCGMc18A* for CGM resistance identified in the present investigation harbors three leucine-rich repeat receptor-like protein kinase (LRR RLK) genes (Manes.18G053900, Manes.18G054000, and Manes.18G054100). LRRs have an important role for recognition specificity and these domains are present in the majority of R proteins which have been described as being effective against disease and insect pests [85,86].

In this study we identify 17 consistent QTL explaining between 1.35%– 45.97% of the phenotypic variance associated with CBSD, CMD, and CGM resistance. To achieve the goal of MAS, further resolution of QTL is necessary and the identification of markers accounting for maximum phenotypic variation. This could include fine-mapping or primers could be designed for specific polymorphisms across the identified QTL regions using the cassava genome sequence, and used to genotype segregating populations or contrasting genotypes, possibly using a bulk segregant analysis approach. Linkage of specific alleles with resistance or susceptibility could then be determined. Interestingly none of the QTL (except *qCGMc9AR*) were consistent across all environments. This has implications for the utility of linked markers derived from these QTL which may not be robust across environments.

## Conclusions

This study identified QTL for multiple disease and pest resistance in cassava. Consistent with earlier studies, we find that root necrosis and foliar symptoms of CBSD are to some extent under different genetic control, and QTL appear to be influenced by genotype-by-environment interactions. We identify a new putative QTL for CBSD root necrosis on chromosome XIV from a variety with approximately 30.25% of its genome of South American origin (AR40-6). In addition, QTL on chromosomes XI and XVIII for CBSD foliar symptoms corroborate the findings from other studies. From our population, in which both parents are thought to possess the *CMD2* locus, we provide further evidence for the existence of two QTL or multi-allelic variants influencing CMD resistance within the locality of the *CMD2* locus. Similarly, the co-localization of our QTL *qCGMc18AR* for CGM resistance with previously identified SSR marker NS346 adds credence to the validity of this QTL. The growing evidence corroborating the effect of certain QTL associated with pest and disease resistance in cassava, and the germplasm in which these QTL exist, helps to progress the application of molecular markers in cassava.

## Supporting information

S1 Fig The climate of experimental sites (Chambezi and Naliendele) in Tanzania. (PPTX)

S2 Fig Description of genetic linkage map of F1 full-sib family of cassava derived from a cross between AR 40–6 and Albert using 2125 SNP markers. The genetic distance between markers is given in centimorgans.
(DOCX)

S1 Table Analysis of variance (ANOVA) for field disease and pest resistance in the AR40-6 x Albert mapping population. (DOCX)

S2 Table Correlation coefficients (r) of CBSD foliar and root necrosis symptoms, foliar CMD and CGM symptoms of the AR40-6 x Albert mapping population across four environments. (DOCX)

S3 Table Trait distributions of full-sib population of AR40-6 x Albert based on pooled mean of four environments. (DOCX)

S4 Table Summary of single nucleotide polymorphism (SNP) markers information of linkage map of AR40-6 x Albert derived clonal F_1_ population. (DOCX)

S5 Table Favourable alleles at QTL flanking markers detected for field disease and pest resistance in the AR40-6 x Albert mapping population. (DOCX)

S6 Table Comparison of previously reported markers with QTL identified in present investigation for disease and pest resistant loci in cassava. (DOCX)

S7 Table Genes underlying the consistent QTL (qCGMc18) for resistance/tolerance to cassava green mite. (XLSX)
