# Supplementary material for: Genetic analysis and QTL mapping for multiple biotic stress resistance in cassava
Source: PLoS One. 2020 Aug 5;15(8):e0236674. doi: 10.1371/journal.pone.0236674 (PMC7406056; doi:10.1371/journal.pone.0236674)
Supplement: S1 Fig — (PPTX) [file pone.0236674.s001.pptx]

## Slide 1
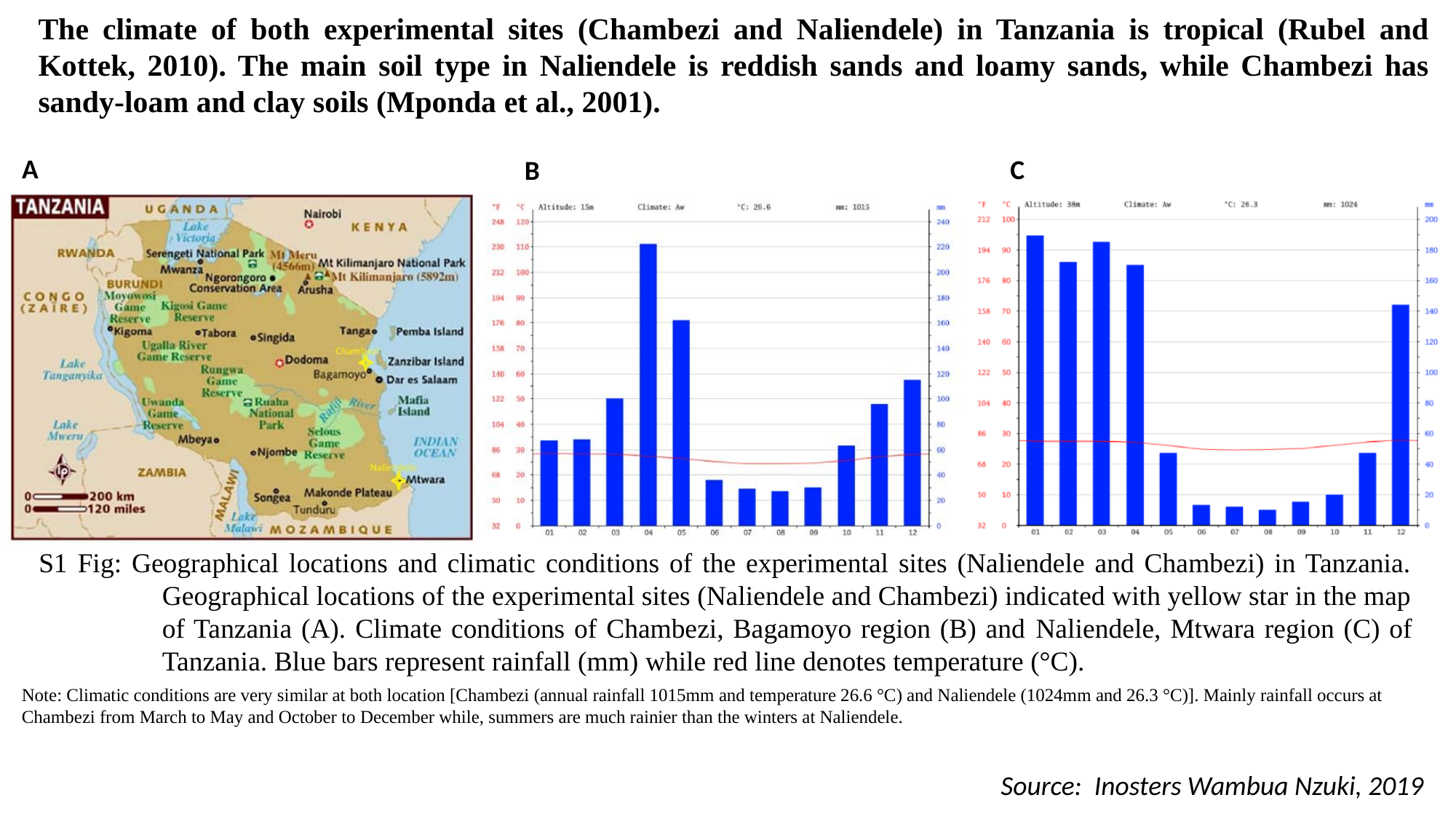

The climate of both experimental sites (Chambezi and Naliendele) in Tanzania is tropical (Rubel and Kottek, 2010). The main soil type in Naliendele is reddish sands and loamy sands, while Chambezi has sandy-loam and clay soils (Mponda et al., 2001).
A
C
B
S1 Fig: Geographical locations and climatic conditions of the experimental sites (Naliendele and Chambezi) in Tanzania. Geographical locations of the experimental sites (Naliendele and Chambezi) indicated with yellow star in the map of Tanzania (A). Climate conditions of Chambezi, Bagamoyo region (B) and Naliendele, Mtwara region (C) of Tanzania. Blue bars represent rainfall (mm) while red line denotes temperature (°C).
Note: Climatic conditions are very similar at both location [Chambezi (annual rainfall 1015mm and temperature 26.6 °C) and Naliendele (1024mm and 26.3 °C)]. Mainly rainfall occurs at Chambezi from March to May and October to December while, summers are much rainier than the winters at Naliendele.
Source: Inosters Wambua Nzuki, 2019
