# Supplementary material for: Genetic analysis and QTL mapping for multiple biotic stress resistance in cassava
Source: PLoS One. 2020 Aug 5;15(8):e0236674. doi: 10.1371/journal.pone.0236674 (PMC7406056; doi:10.1371/journal.pone.0236674)
Supplement: S2 Fig — The genetic distance between markers is given in centimorgans. (DOCX) [file pone.0236674.s002.docx]

**S2 Fig.** Description of genetic linkage map of F_1_ full-sib family of cassava derived from a cross between AR 40-6 and Albert using 2125 SNP markers. The genetic distance between markers is given in centimorgans.
