# Supplementary material for: Genetic analysis and QTL mapping for multiple biotic stress resistance in cassava
Source: PLoS One. 2020 Aug 5;15(8):e0236674. doi: 10.1371/journal.pone.0236674 (PMC7406056; doi:10.1371/journal.pone.0236674)
Supplement: S1 Table — (DOCX) [file pone.0236674.s003.docx]

**S1 Table:**  Analysis of variance (ANOVA) for field disease and pest resistance in the AR40-6 x Albert mapping population

| **Traits** | **Scoring/ analysis stage** | **Mean squares** | | | | | | | | | | | | | | | | | | | | |
| --- | --- | --- | --- | --- | --- | --- | --- | --- | --- | --- | --- | --- | --- | --- | --- | --- | --- | --- | --- | --- | --- | --- |
|  |  | **Cham 13** | | |  | **Cham 14** | | |  | **Nal 13** | | |  | **Nal 14** | | |  | **Combined** | | | | |
|  |  | Gen | Rep | Block (Rep) |  | Gen | Rep | Block (Rep) |  | Gen | Rep | Block (Rep) |  | Gen | Rep | Block (Rep) |  | Env | Rep (Env) | Blocks (Env*Rep) | Gen | Gen*Env |
| CBSDF | 3 MAP | 0.475* | 0.018 | 0.279 |  | 0.502** | 0.007 | 0.255 |  | 0.407** | 0.116 | 0.098 |  | 0.367** | 0.011 | 0.111 |  | 2.260** | 0.038 | 0.185 | 1.235** | 0.172 |
|  | 6 MAP | 0.527** | 2.879** | 0.311 |  | 0.559** | 2.456** | 0.313 |  | 0.393** | 0.159 | 0.210* |  | 0.450** | 0.128 | 0.205** |  | 11.36** | 1.406** | 0.259* | 1.155** | 0.258** |
| CMD | 3 MAP | 1.290** | 0.013 | 0.535 |  | 1.361** | 0.003 | 0.472 |  | 2.277** | 0.143 | 0.449** |  | 2.263** | 0.110 | 0.323* |  | 2.734** | 0.067 | 0.445** | 5.091** | 0.701** |
|  | 6 MAP | 1.108** | 0.186 | 0.491* |  | 1.160** | 0.433 | 0.465* |  | 1.247** | 0.665* | 0.121 |  | 1.252** | 0.003 | 0.080 |  | 0.032 | 0.321 | 0.289** | 3.186** | 0.527** |
| CGM | 3 MAP | 0.242** | 1.389** | 0.132 |  | 0.250** | 1.727** | 0.108 |  | 0.141* | 0.519* | 0.075 |  | 0.154* | 0.364 | 0.081 |  | 0.498** | 1.000** | 0.099 | 0.336** | 0.120 |
|  | 6 MAP | 0.255** | 0.466** | 0.090* |  | 0.254** | 0.360* | 0.080 |  | 0.409** | 0.211 | 0.215 |  | 0.419** | 0.042 | 0.257 |  | 8.818** | 0.270* | 0.160* | 0.804** | 0.178** |
| RNS | Harvesting | 1.863** | 2.329** | 0.718 |  | 1.054** | 4.227** | 0.571 |  | 0.594** | 0.223 | 0.260 |  | 0.569** | 1.115* | 0.359 |  | 58.76** | 7.213** | 0.477 | 1.386** | 0.898** |
| RNA% | Harvesting | - | - |  |  | 300.1** | 21.68 | 103.23 |  | 111.3** | 11.48 | 31.75 |  | 152.0** | 175.7 | 60.95 |  | 157.6* | 69.61* | 65.31 | 352.9** | 105.3** |

CBSD: Cassava Brown Streak Disease; CMD: Cassava Mosaic Disease; CGM: Cassava Green Mite; RNS: Root Necrosis Score (Scale 1-5); RNA%: Root Necrosis Area (%); 3 MAP and 6 MAP: 3 and 6 Months after Planting stage, respectively. Note: * and ** represent significant at P < 0.05 and P < 0.01, respectively.
