# Supplementary material for: Genetic analysis and QTL mapping for multiple biotic stress resistance in cassava
Source: PLoS One. 2020 Aug 5;15(8):e0236674. doi: 10.1371/journal.pone.0236674 (PMC7406056; doi:10.1371/journal.pone.0236674)
Supplement: S2 Table — (DOCX) [file pone.0236674.s004.docx]

**S2 Table: Correlation coefficients (*r*) of CBSD foliar and root necrosis symptoms, foliar CMD and CGM symptoms of the AR40-6 x Albert mapping population across four environments**

**S2A:** Correlation coefficients (*r*) of CBSD root necrosis and foliar of the AR40-6 x Albert mapping population across four environments

| **Environments** | **CHA13** | **CHA14** | **NAL13** | **NAL14** |
| --- | --- | --- | --- | --- |
| **CHA13** | 1.000 | 0.381** | 0.188* | 0.108 |
| **CHA14** | 0.988**  (0.967**) | 1.000 | 0.095  (0.164) | 0.107  (0.153) |
| **NAL13** | 0.438**  (0.217*) | 0.418**  (0.230*) | 1.000 | 0.384**  (0.452**) |
| **NAL14** | 0.395**  (0.187*) | 0.375**  (0.199*) | 0.982** (0.967**) | 1.000 |

Note: *r* values based on CBSD root necrosis [root necrosis (RN) data; RN scale and RN per cent data are in open and parentheses, respectively] are presented above diagonal while CBSD foliar symptom [CBSD foliar data: CBSD at 3 MAP and CBSD at 6 MAP are in open and parentheses, respectively] are presented below diagonal. All *r* values with * and ** were significant at *P* = 0.05 and 0.01, respectively. CHA13, CHA14, NAL13 and NAL14 represent environment Chambezi (CHA) in the year of 2013 (13) and 2014 (14) and Naliendele (NAL) in the year of 2013 (13) and 2014 (14), respectively.

**S2B:** Correlation coefficients (*r*) of CMD and CGM of the AR40-6 x Albert mapping population across four environments

| **Environments** | **CHA13** | **CHA14** | **NAL13** | **NAL14** |
| --- | --- | --- | --- | --- |
| **CHA13** | 1.000 | 0.987**  (0.983**) | 0.433**  (0.355**) | 0.428**  (0.359**) |
| **CHA14** | 0.983**  (0.949**) | 1.000 | 0.428**  (0.357*) | 0.432**  (0.363*) |
| **NAL13** | 0.121  (0.242**) | 0.114  (0.284**) | 1.000 | 0.989**  (0.991**) |
| **NAL14** | 0.110  (0.227*) | 0.103  (0.281**) | 0.968**  (0.971**) | 1.000 |

Note: *r* values based on CMD [CMD data: CMD at 3 MAP and CMD at 6 MAP are in open and parentheses, respectively] are presented above diagonal while CGM symptom [CGM data: CGM at 3 MAP and CGM at 6 MAP are in open and parentheses, respectively] are presented below diagonal. All *r* values with * and ** were significant at *P* = 0.05 and 0.01, respectively
