# Supplementary material for: Genetic analysis and QTL mapping for multiple biotic stress resistance in cassava
Source: PLoS One. 2020 Aug 5;15(8):e0236674. doi: 10.1371/journal.pone.0236674 (PMC7406056; doi:10.1371/journal.pone.0236674)
Supplement: S3 Table — (DOCX) [file pone.0236674.s005.docx]

**S3 Table:** Trait distributions of full-sib population of AR40-6 x Albert based on pooled mean of four environments (crop seasons 2013 and 2014 at Chambezi and Naliendele, Tanzania)

| **Traits** | **Scoring/ analysis stage** | **Mean**  **(±SE)** | **Range** | | **GCV** | **PCV** | **Skewness** | **Kurtosis** | **h^2^ (%)** |
| --- | --- | --- | --- | --- | --- | --- | --- | --- | --- |
|  |  |  | **Min** | **Max** |  |  |  |  |  |
| CBSDF | 3 MAP | 1.265±0.276 | 1.00 | 2.65 | 18.92 | 28.89 | 1.249 | 0.495 | 42.91 |
|  | 6 MAP | 1.390±0.352 | 1.00 | 2.75 | 14.46 | 29.10 | 0.688 | -0.581 | 24.70 |
| CMD | 3 MAP | 2.049±0.445 | 1.00 | 4.00 | 37.69 | 43.49 | 0.411 | -1.139 | 75.16 |
|  | 6 MAP | 1.865±0.320 | 1.00 | 3.00 | 31.17 | 35.57 | 0.335 | -0.914 | 76.79 |
| CGM | 3 MAP | 1.141±0.257 | 1.00 | 2.40 | 11.72 | 25.40 | 2.076 | 3.085 | 21.29 |
|  | 6 MAP | 1.414±0.276 | 1.00 | 2.25 | 14.93 | 24.60 | 0.516 | -0.665 | 36.84 |
| RNS | Harvesting | 1.856±0.376 | 1.00 | 3.14 | 14.29 | 24.78 | 0.566 | -0.315 | 33.26 |
| RNA% | Harvesting | 12.91±6.470 | 4.27 | 61.20 | 29.04 | 57.94 | 2.715 | 11.140 | 25.12 |

CBSD: Cassava Brown Streak Disease; CMD: Cassava Mosaic Disease; CGM: Cassava Green Mite; RNS: Root Necrosis Score (Scale 1-5); RNA%: Root Necrosis Area (%); 3 MAP and 6 MAP: 3 and 6 Months after Planting stage, respectively; SE: Standard error; GCV: Genotypic Coefficient of Variation; PCV: Phenotypic Coefficient of Variation; h^2^: Broad sense heritability
