# Supplementary material for: Genetic analysis and QTL mapping for multiple biotic stress resistance in cassava
Source: PLoS One. 2020 Aug 5;15(8):e0236674. doi: 10.1371/journal.pone.0236674 (PMC7406056; doi:10.1371/journal.pone.0236674)
Supplement: S4 Table — (DOCX) [file pone.0236674.s006.docx]

**S4 Table:** Summary of single nucleotide polymorphism (SNP) markers information of linkage map of AR40-6 x Albert derived clonal F_1_ population

| **Chromosome Number** | **Number of Mapped SNPs Markers** | **Length of linkage group (cM)** | **Average marker**  **interval (cM)** |
| --- | --- | --- | --- |
|  |  |  |  |
| I | 137 | 92.6 | 0.68 |
| II | 134 | 118.1 | 0.88 |
| III | 144 | 112.2 | 0.78 |
| IV | 121 | 110.1 | 0.91 |
| V | 157 | 103.6 | 0.66 |
| VI | 161 | 121.9 | 0.76 |
| VII | 107 | 100.6 | 0.94 |
| VIII | 76 | 103.0 | 1.36 |
| IX | 158 | 109.9 | 0.70 |
| X | 115 | 51.6 | 0.45 |
| XI | 145 | 113.8 | 0.78 |
| XII | 62 | 49.8 | 0.80 |
| XIII | 86 | 99.3 | 1.15 |
| XIV | 159 | 104.2 | 0.66 |
| XV | 139 | 117.9 | 0.85 |
| XVI | 45 | 76.9 | 1.71 |
| XVII | 85 | 77.8 | 0.92 |
| XVIII | 94 | 66.7 | 0.71 |
| **Mean** | **118.06** | **96.11** | **0.81** |
| **Total** | **2125** | **1730** |  |
