# Supplementary material for: Genetic analysis and QTL mapping for multiple biotic stress resistance in cassava
Source: PLoS One. 2020 Aug 5;15(8):e0236674. doi: 10.1371/journal.pone.0236674 (PMC7406056; doi:10.1371/journal.pone.0236674)
Supplement: S5 Table — (DOCX) [file pone.0236674.s007.docx]

**S5 Table:** Favourable alleles at QTL flanking markers detected for field disease and pest resistance in the AR40-6 x Albert mapping population

| **Trait** | **Scoring/ analysis stage** | **QTL** | **Chromosome** | **Flanking Markers** | **Favourable Allele** |
| --- | --- | --- | --- | --- | --- |
| CBSD-Foliar | *3 MAP* | *qCBSDFc11AR* | XI | 18700194 | T/T |
|  |  |  |  | 18472436 | T/A |
|  | *6 MAP* | *qCBSDFc18AR* | XVIII | 10068641 | G/G |
|  |  |  |  | 10924641 | A/A |
| CBSD- Necrosis* | *Harvesting* | *qCBSDRNSc14AR* | XIV | 12107960 | C/C |
|  |  |  |  | 13964594 | T/T |
| CBSD- Necrosis** | *Harvesting* | *qCBSDRNAc8AR* | VIII | 5883289 | T/T |
|  |  |  |  | 4660806 | G/G |
|  |  | *qCBSDRNAc14AR* | XIV | 12866832 | A/A |
|  |  |  |  | 13596996 | G/T |
|  |  | *qCBSDRNAc18AR* | XVIII | 6433344 | T/T |
|  |  |  |  | 6501916 | C/C |
| CMD | *3 MAP* | *qCMDc9AA* | IX | 16028318 | A/G |
|  |  |  |  | 16697056 | G/G |
|  |  | *qCMDc10.1AA* | X | 3711657 | A/A |
|  |  |  |  | 3562661 | A/A |
|  |  | *qCMDc12.1AA* | XII | 5330694 | A/A |
|  |  |  |  | 5237169 | T/T |
|  | *6 MAP* | *qCMDc10.2AA* | X | 17833540 | T/T |
|  |  |  |  | 16727950 | G/G |
|  |  | *qCMDc12.2AA* | XII | 6745505 | G/G |
|  |  |  |  | 5224029 | T/C |
|  |  | *qCMDc12.1AA* | XII | 5330694 | A/A |
|  |  |  |  | 5237169 | T/T |
| CGM | *3 MAP* | *qCGMc5AR* | V | 2287186 | T/C |
|  |  |  |  | 1801930 | A/A |
|  |  | *qCGMc9AR* | IX | 984855 | G/G |
|  |  |  |  | 1938190 | G/G |
|  |  | *qCGMc13AR* | XIII | 16122724 | G/G |
|  |  |  |  | 16635048 | C/C |
|  |  | *qCGMc18AR* | XVIII | 3701705 | T/T |
|  |  |  |  | 3265545 | G/G |
|  | *6 MAP* | *qCGMc16AR* | XVI | 1619079 | C/T |
|  |  |  |  | 15707994 | G/T |

Note: *and ** denote CBSD root necrosis assessed based on visual scale (1-5) and actual (%) necrotic area quantified by ImageJ program, respectively
