# Supplementary material for: Genetic analysis and QTL mapping for multiple biotic stress resistance in cassava
Source: PLoS One. 2020 Aug 5;15(8):e0236674. doi: 10.1371/journal.pone.0236674 (PMC7406056; doi:10.1371/journal.pone.0236674)
Supplement: S6 Table — (DOCX) [file pone.0236674.s008.docx]

**S6 Table.** Comparison of previously reported markers with QTLs identified in present investigation for disease and pest resistant loci in cassava.

| **Trait** | **Locus Name** | **SSR markers** | **Reference(s)** | **Chromosome (ref. genome V6.1)** | **Chromosomal position (ref. genome V6.1)** | **Length (bp)** | **Cassava gene carrying marker** | **Marker Interval of the present investigation (ref. genome V5.1)** | **QTL in Present study** |
| --- | --- | --- | --- | --- | --- | --- | --- | --- | --- |
| CMD | *CMD1* | SSR 40 | Fregene and Puonti-Kaerlas, 2002 | 1 | Chr.01:32767620..32767858 | 239 | Manes.01G251600  (5^th^ intron) | SNP_24421896-SNP_25660182 | - |
|  | *CMD2* | SSRY 28 | Akano et al., 2002; Lokko et al., 2005; Okogbenin et al., 2012 | 12 | Chr.12:7541033..7541198 | 166 | Manes.12G074000  (5^th^ exon & 4^th^ intron) | SNP_6745505 - SNP_7787788 | *qCMDc12.2* |
|  |  | SSR NS 158 | Okogbenin et al., 2007 | 12 | Chr.12:7731640..7731816 | 177 | Manes.12G074900  (3^rd^ intron) | SNP_6745505 - SNP_7787788 | *qCMDc12.2* |
|  |  | SSR NS 169 | Okogbenin et al., 2007 | 12 | Chr.12:7731640..7731970 | 331 | Manes.12G074900  (3^rd^ intron & 4^th^ exon) | SNP_6745505 - SNP_ 7787788 | *qCMDc12.2* |
|  | *CMD3* | NS 198 | Okogbenin et al., 2012 | 12 | Chr.12:1353173..1353364 | 192 | Manes.12G016800  (2^nd^ intron) | SNP_1106091 - SNP_ 1275959 | - |
| CBSD root necrosis* | - | S11-19872319, S11- 23751929, S11- 22909579, S11-22909532, S11-23228224 | Kawuki et al., 2016 | 11 | Chr.11:19872319..23751929 | - | - | - | - |
| CGM | - | SSR NS 346 | Ceballos et al., 2010; Macea-Choperena et al. 2012 | 18 | Chr.18:4143253..4143542 | 290 | Manes.18G049600  (3^rd^ exon & 3^rd^ intron) | SNP_3265545-SNP_3701705 | *qCGMc18* |
|  | - | SSR NS 1009** | Ceballos et al., 2010 | 14 | - | - | - | - | - |
|  | - | SSR NS 1099 | Macea-Choperena et al. 2012 | 18 | Chr.18:1301130..1301345 | 216 | Manes.18G016300  (15^th^ intron) | SNP_9696899- SNP_11427743 | - |

* QTL associated with CBSD root necrosis tagged with SNP markers; **Only sequence of forward primer was detected at chromosome XIV in Cassava reference genome v6.1
